# XB130 promotes bronchioalveolar stem cell and Club cell proliferation in airway epithelial repair and regeneration

**DOI:** 10.18632/oncotarget.5062

**Published:** 2015-09-03

**Authors:** Hiroaki Toba, Yingchun Wang, Xiaohui Bai, Ricardo Zamel, Hae-Ra Cho, Hongmei Liu, Alonso Lira, Shaf Keshavjee, Mingyao Liu

**Affiliations:** ^1^ Latner Thoracic Surgery Research Laboratories, Toronto General Research Institute, Universal Health Network, Toronto, ON, Canada; ^2^ Department of Surgery, Faculty of Medicine, University of Toronto, Toronto, ON, Canada; ^3^ Institute of Medical Science, Faculty of Medicine, University of Toronto, Toronto, ON, Canada

**Keywords:** naphthalene, small airway injury and repair, bronchioalveolar duct junction, PI3K/Akt signaling, transgenic mice

## Abstract

Proliferation of bronchioalveolar stem cells (BASCs) is essential for epithelial repair. XB130 is a novel adaptor protein involved in the regulation of epithelial cell survival, proliferation and migration through the PI3K/Akt pathway. To determine the role of XB130 in airway epithelial injury repair and regeneration, a naphthalene-induced airway epithelial injury model was used with *XB130* knockout (KO) mice and their wild type (WT) littermates. In *XB130* KO mice, at days 7 and 14, small airway epithelium repair was significantly delayed with fewer number of Club cells (previously called Clara cells). CCSP (Club cell secreted protein) mRNA expression was also significantly lower in KO mice at day 7. At day 5, there were significantly fewer proliferative epithelial cells in the KO group, and the number of BASCs significantly increased in WT mice but not in KO mice. At day 7, phosphorylation of Akt, GSK-3β, and the p85α subunit of PI3K was observed in airway epithelial cells in WT mice, but to a much lesser extent in KO mice. Microarray data also suggest that PI3K/Akt-related signals were regulated differently in KO and WT mice. An inhibitory mechanism for cell proliferation and cell cycle progression was suggested in KO mice. XB130 is involved in bronchioalveolar stem cell and Club cell proliferation, likely through the PI3K/Akt/GSK-3β pathway.

## INTRODUCTION

The lung epithelium provides an important defense mechanism against various infectious and noxious substances in the air. Repair and regeneration of the epithelium after injury is essential for normal function. Improper tissue repair and remodeling leads to lung diseases, including chronic obstructive pulmonary disease, asthma and pulmonary fibrosis [1].

In the bronchioles, epithelial cells consist of both ciliated cells and non-ciliated Club cells (previously called Clara cells). Club cells are much more abundant, accounting for 70–90% of epithelial cells in mice [2]. Naphthalene is an environmental pollutant that specifically ablates Club cells [3], and is commonly used as a model to study small airway injury and repair [4, 5]. Following naphthalene injury, a small subset of surviving Club cells, variant Club cells, is found at the bronchioalveolar duct junctions (BADJs) [6]. They proliferate and differentiate into Club cells, which leads to the renewal of the small airway epithelium [7]. These cells have been proposed as bronchioalveolar stem cells (BASCs), located at the BADJs [8]. Under certain circumstances, BASCs also contribute to the regeneration of alveolar cells [8], thus playing important roles in the repair and regeneration of terminal bronchioles and alveoli.

Several intracellular signal transduction pathways have been suggested to regulate the proliferation and differentiation of BASCs. The PI3K pathway has been proposed as a critical regulator of BASC expansion [9]. Using a transgenic approach, Tiozzo and co-workers selectively deleted Pten, a negative regulator of the PI3K pathway, in lung epithelial cells. This deletion increased BASCs at the BADJs, and conferred a selective advantage to naphthalene injury in these cells [10]. Further exploration of the role of molecules in the PI3K pathway may enhance our knowledge of how bronchial epithelial repair and regeneration is regulated.

XB130 (also called AFAP1L2, for actin filament associated protein 1 like 2) is a novel adaptor protein for intracellular signal transduction [11]. It regulates cell cycle progression, prevents cell death, and promotes cell migration through its binding with p85α, the regulatory subunit of PI3K, and subsequently activates the PI3K/Akt signaling pathway [12–14]. XB130 protein is found in various epithelial cells in the stomach [15] esophagus [16] and thyroid [17] and its potential as a signal transduction protein has been investigated *in vitro* [18, 19]. Using *XB130* knockout (KO) mice, it has been shown that XB130 deficiency affects tracheal epithelial differentiation during airway repair [20]. Nicotine-derived nitrosamine ketone (NNK) is the most potent carcinogen among cigarette smoking components. Recently, it has been shown that XB130 mediates NNK-induced migration of human bronchial airway epithelial cells [21]. However, little is known about the function of XB130 in bronchial airways *in vivo*. We hypothesized that the absence of XB130 leads to greater susceptibility to severe injury, and delays epithelial cell repair through the PI3K/Akt pathway. This hypothesis was tested with the naphthalene-induced airway epithelial injury model using *XB130* knock out (KO) mice.

## RESULTS

### XB130 is highly expressed in the bronchial epithelium of normal mouse lung

**Figure 1:**
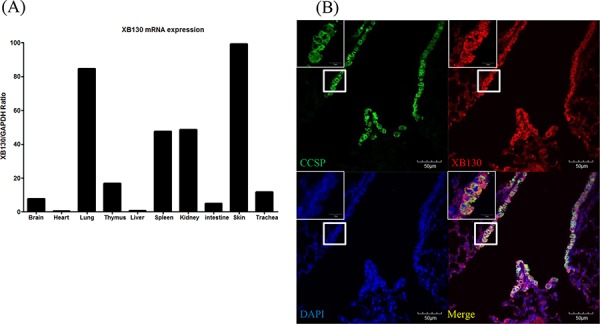
Expression of XB130 in murine small airway epithelial cells **A.** The *XB130* mRNA level of normal mouse lung was relatively high compared to those of other organs studied, as determined by RT-PCR. **B.** Immunofluorescence studies show XB130 expression (XB130+, red) in Club cells (CCSP+, green) in the small airway epithelium of normal mouse lung.

We examined the *XB130* mRNA expression in various mouse organ tissues of WT mice. The *XB130* mRNA expression in mouse lung was relatively higher compared to other organs (Figure 1A). *XB130* was strongly expressed in the cytoplasm of Club cells (Figure 1B), and as expected, no expression of *XB130* was found in *XB130* KO mice (data not shown). *XB130* was also found in both type I and type II alveolar epithelial cells, but at a much lower level than in the airway epithelial cells (data not shown).

### XB130 deficiency leads to delay of airway epithelial repair

Body weight loss is known to be a good marker of the severity of naphthalene-induced injury [22]. There was no significant difference in weight loss between WT and KO mice (Figure E1 in the online data supplement).

**Figure 2:**
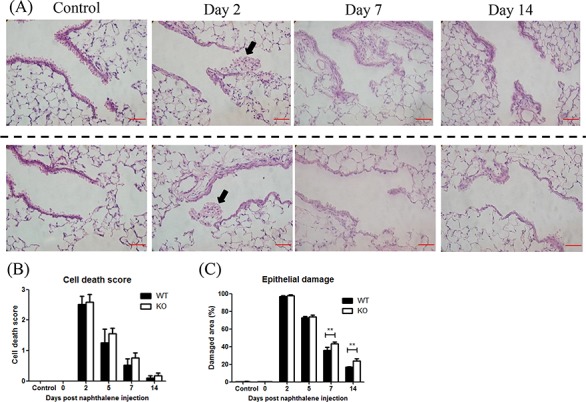
XB130 deficiency delayed the repair process after naphthalene-induced small airway epithelial damage **A.** Naphthalene-induced small airway injury and repair. Representative histological changes of lung tissues at different stages of injury and repair from wild type (WT) and *XB130* knockout (KO) mice. At day 2 after naphthalene treatment, many dead cells (arrow) were observed in the airway lumen in both groups. Scale Bars = 50 μm. **B.** The cell death scores did not show significant difference between two groups. **C.** At day 7 and 14, damaged area of airway epithelium was significantly larger in KO mice (**p < 0.01).

Under the control condition (day 2 after corn oil without naphthalene), the morphology of lung tissue is indistinguishable between the KO and WT groups (Figure 2A), suggesting that XB130 ablation has no effect on lung development. At day 2 after naphthalene treatment, many dead cells were detached from the basement membrane and sloughed into the airway lumen, and the surface of the airway epithelium was covered by a thin layer of survival cells, in both WT and *XB130* KO mice (Figure 2A). The bronchial airway epithelium gradually recovered after the injury at days 7 and 14; at day 14, the morphology of bronchial airway epithelium in the WT mice appears similar to that of the control (Figure 2A). The cell death scores did not show significant differences between the two groups (Figure 2B). However, at days 7 and 14, the damaged areas of airway epithelium were significantly larger in KO mice (Figure 2A and 2C), suggesting a delay of bronchial airway epithelial repair.

### XB130 deficiency leads to reduced bronchial epithelial cell proliferation

**Figure 3:**
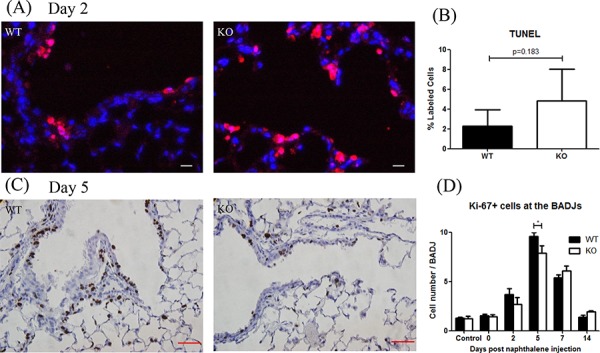
XB130 deficiency reduced cell proliferation during small airway epithelial repair **A, B.** At day 2 after naphthalene treatment, apoptotic cells (red) were observed in both groups without significant difference. Scale Bars = 20 μm. **C.** At day 5, the number of proliferative (Ki-67+, brown) epithelial cells was less in KO mice. Scale Bars = 50 μm. **D.** Ki67+ cells were quantified as an index for cell proliferation at different time points. At day 5, the number of Ki67+ cells in KO mice was significantly less than that in WT mice (*p < 0.05).

At day 2 after naphthalene treatment, most of the detached cells were apoptotic (data not shown). To assess the extent of cell death in airway lumen, we counted attached TUNEL+ cells in the airway wall at the BADJs (Figure 3A). There was no significant difference between the two groups (Figure 3B). At day 5, the number of Ki-67+ cells, a marker for cell proliferation (Figure 3C), was significantly lower in KO mice (Figure 3D).

**Figure 4:**
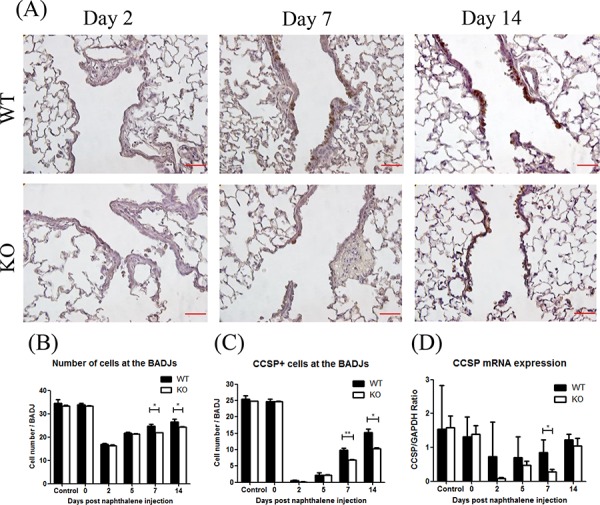
XB130 deficiency reduced Club cell proliferation during small airway epithelial repair **A.** The number of Club cells was determined by immunohistochemistry staining. Less staining at day 7 and 14 was noted in KO mice. Scale Bars = 50 μm. At day 7 and 14 after naphthalene treatment, the number of total epithelial cells **B.** and Club cells (CCSP+) **C.** was significantly less at the bronchioalveolar duct junctions (BADJ) in KO mice. **D.** The CCSP mRNA expression was also significantly lower in KO mice at day 7. (*p < 0.05).

At days 7 and 14 after naphthalene treatment, the total number of airway epithelial cells quantified from H&E staining at the BADJ were significantly less in the KO mice (Figure 4B). The number of CCSP+ cells (a marker for Club cells) [23, 24] was also significantly lower in the KO mice (Figure 4A, 4C). Furthermore, the CCSP mRNA expression was significantly lower in KO mice at day 7 (Figure 4D). These results suggest that XB130 may play an important role in airway epithelial cell regeneration at the BADJs after injury, especially by affecting Club cell proliferation.

### XB130 deficiency did not affect ciliated cells after injury

**Figure 5:**
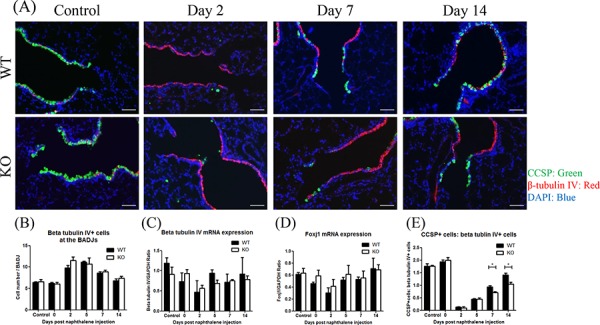
XB130 deficiency did not affect ciliated cells during small airway epithelial repair **A.** Immunofluorescence study shows a dynamic change for Club cells (CCSP+, green) and ciliated epithelial cells (β-tubulin IV+, red) after naphthalene challenge at the BADJs. Scale Bars = 50 μm. **B.** At day 2 and 5, the number of ciliated epithelial cells (β-tubulin IV+) increased at the BADJs in both groups. **C, D.** The mRNA expressions of β-tubulin IV and Foxj1 (another ciliated cell marker) did not change over time. **E.** At day 7 and 14 after naphthalene treatment, the ratio of Club cells to ciliated epithelial cells was significantly lower in KO mice. (*p < 0.05).

In general, after Club cell ablation, ciliated cells are well known to cover the denuded basement membrane of the injured airway epithelium through cell spreading and migration [5, 25]. To determine the role of XB130 on the behavior of both Club cells and ciliated cells during airway injury and repair, CCSP and β-tubulin IV (ciliated cell marker) [26, 27] double staining was performed (Figure 5). Under the control condition, the small airway was primarily covered with CCSP+ cells (Club cells, green). Two days after naphthalene treatment, the BADJ area was mainly covered by β-tubulin IV+ cells (ciliated cells, red). At days 7 and 14, the number of CCSP+ cells increased at the BADJs in the WT mice and to a lesser extent in the KO mice (Figure 5A). At days 2 and 5, the number of β-tubulin IV+ cells increased at the BADJs and then gradually decreased in both groups (Figure 5B). The mRNA expression of β-tubulin IV and Foxj1 (another ciliated cell marker) [26, 27] was also not significantly different between the two groups (Figure 5C and 5D). On the other hand, the ratio of CCSP+ cells to β-tubulin IV+cells was significantly lower in KO mice at days 7 and 14 (Figure 5E).

**Figure 6:**
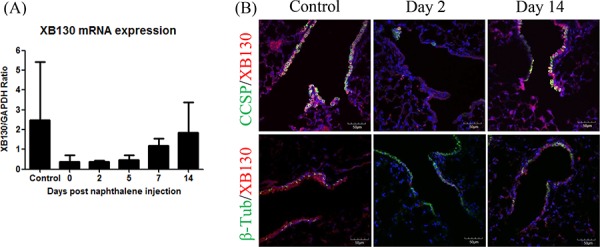
Expression of XB130 in Club cells but not in ciliated cells **A.** *XB130* gene expression changes after naphthalene treatment in the lung tissues of WT mice, determined by RT-PCR. **B.** XB130 (red) was co-stained with CCSP (for Club cells, green), or with β-tubulin IV (for ciliated cells, green). The disappearance and reappearance of XB130 during injury and repair was associated with Club cells.

In WT mice, the *XB130* mRNA level was reduced in the lung tissue after naphthalene administration, and gradually recovered over time (Figure 6A). XB130 was co-stained with CCSP (for Club cells; Figure 6B Top), or with β-tubulin IV (for ciliated cells; Figure 6B Bottom). At day 2, when the small airway was mainly covered with ciliated cells, no XB130 expression was noted, whereas at day 14, the recovery of Club cells was associated with XB130 expression (Figure 6B). These results suggest that XB130 deficiency mainly affects the function of Club cells, not ciliated cells.

### XB130 deficiency slows down proliferation of bronchioalveolar stem cells

**Figure 7:**
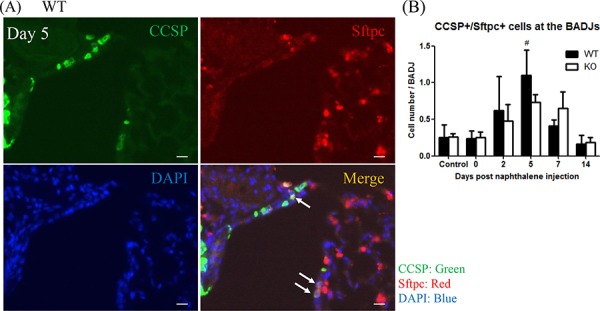
XB130 deficiency reduced bronchioalveolar stem cell proliferation during small airway epithelial repair **A.** Immunofluorescence staining shows the localization of bronchioalveolar stem cells (BASCs; arrows, CCSP+/Sftpc+) at the BADJs at day 5 after naphthalene treatment in WT mice. Scale Bars = 20 μm. **B.** At day 5, the number of BASCs was significantly increased in WT mice compared to control, day 0 and day 14 groups. The number of BASCs in KO mice was not significantly increased. (#*p* < 0.05).

We further tested whether XB130 deficiency also affects the local stem cells (variant Club cells, or BASCs). The BASCs were determined with co-IF staining for CCSP and Surfactant protein C (Sftpc) double positive cells at the BADJs [8, 28, 29]. Among CCSP+ cells, a few of them were CCSP+/Sftpc+ cells (Figure 7A, white arrows). At day 5, the number of BASCs significantly increased compared to both days 0 and 14 and the control group in WT mice (Figure 7B). In KO mice, the number of BASCs also increased at days 5 and 7 but was not significantly different from other time points. Thus, XB130 deficiency reduced the proliferation of BASCs at the BADJs.

### XB130 deficiency reduced phosphorylation of proteins on PI3K/AKT pathway during airway epithelial repair

**Figure 8:**
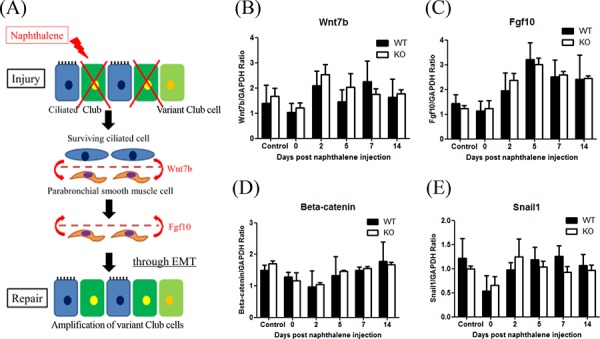
XB130 deficiency did not affect expression of genes related to paracrine-induced epithelial mesenchymal transition (EMT) during small airway epithelial repair **A.** The Schema shows a paracrine regulation between surviving variant Club cells (as local stem cells) and parabronchial smooth muscle cells that promote EMT during airway epithelial repair after naphthalene challenge. **B–E.** The mRNA expression levels on 4 related genes (Wnt7b, Fgf10, β-catenin and Snail1) were not significantly different between WT and KO mice.

Volckaert *et al*. showed that crosstalk between epithelial cells and parabronchial smooth muscle cells PSMCs contributed to airway epithelial repair after naphthalene-induced injury through epithelial mesenchymal transition (EMT) [30]. After naphthalene challenge, surviving ciliated cells may stimulate PSMCs through Wnt7b, to induce the release of FGF10, which then stimulates variant Club cells (as local stem cells), initiating the epithelial repair process through a transient EMT (Figure 8A). To determine whether XB130 is involved in these processes, we examined the mRNA expression of EMT-related genes (Wnt7b, Fgf10, β-catenin and Snail1) with quantitative RT-PCR. There were no significant differences between WT and KO mice during the experimental period (Figure 8B–8E).

**Figure 9:**
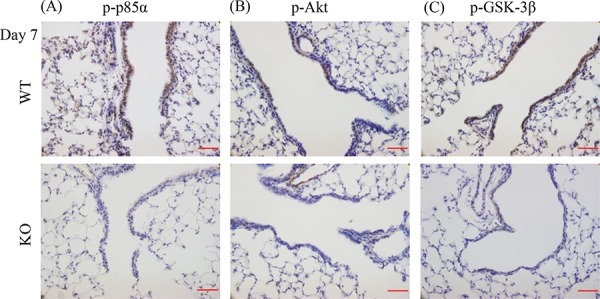
XB130 deficiency reduced phosphorylation of molecules on PI3K/Akt cascade during small airway epithelial repair **A–C.** Immunohistochemistry staining (brown) for p-p85α (regulatory submit of PI3K), p-Akt and p-GSK-3β at day 7 after naphthalene treatment in airway epithelial cells was seen strongly in WT mice but not in KO mice. Scale Bars = 50 μm.

We have previously shown a specific binding interaction between XB130 and the p85α subunit of PI3K [12], which affected cell proliferation and survival via Akt and multiple down-stream signals, including GSK-3β [13]. At day 7 after naphthalene treatment, phosphorylation of p85α, Akt and GSK-3β were observed in the small airways of WT mice, but was significantly less in KO mice (Figure 9A–9C).

### XB130 deficiency affected naphthalene-induced gene expression during lung repair

**Figure 10:**
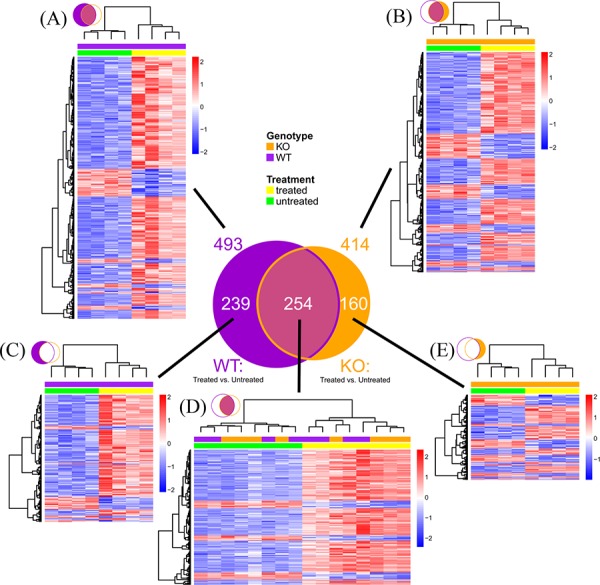
Microarray mouse lung gene expression profile 7 days after naphthalene or no treatment Venn diagram summarizes number of genes with significant expression differences (Fold ≥ 1.5, FDR ≤ 0.05) at day 7 between naphthalene treated and untreated mice (*n* = 4 per group). Purple circle: WT mice. Orange circle: KO mice. **A–E.** Hierarchical clustering heatmaps show relative expression differences for each gene represented in the Venn diagram. Rows represent genes. Columns represent mouse samples. Gene expression is z-score normalized across rows, where low, mean, and high expression levels are represented by blue, white and red, respectively. Color bars above columns indicate genotype (KO: orange, WT: purple) and naphthalene treatment (treated: yellow, untreated: green). (A) All significant genes in WT. (B) All significant genes in KO. (C) Significant genes in WT but not significant in KO. (D) Genes significant in both WT and KO. (E) Significant genes in KO but not significant in WT.

To further determine the potential mechanisms by which XB130 deficiency affects airway repair, we performed microarray studies with RNA extracted from lung tissue 7 days after naphthalene challenge and from normal, untreated mice as controls. Seven days was chosen because multiple differences (e.g., epithelial damage, CCSP+ cell number, CCSP gene expression, and phosphorylation of proteins in PI3K/Akt pathways) are found at this time point. In the untreated lungs, only the expression of 10 genes was significantly different in the KO mice compared to the WT mice. Of these, the top one is *XB130*, as is expected for an *XB130* knock out. Seven days after the naphthalene challenge, 493 genes had significant expression differences compared to the untreated WT group (Figure 10A), and 414 genes compared to the KO group (Figure 10B). Of these, there were 254 gene transcripts in common between the KO and WT groups (Figure 10D). A larger proportion of genes showed increased expression in the naphthalene-treated group, especially in the WT-specific genes (Figure 10C) and those commonly regulated in both WT and KO groups (Figure 10D). By contrast, in the KO-specific group, the number of up-regulated genes is similar to that of the down-regulated genes (Figure 10E).

**Figure 11:**
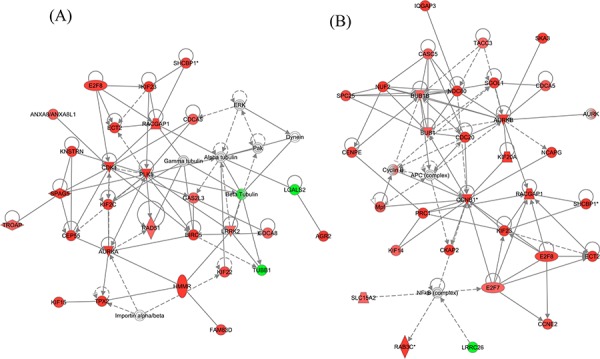
The top IPA generated interaction network of naphthalene-induced genes in (A) WT and (B) KO mice These networks are enhanced for Cell Cycle, Cellular Assembly and Organization, DNA Replication, Recombination, and Repair functions. Red: up-regulation (treated relative to untreated). Green: down-regulation. Gray: does not meet cutoff values for significance. White: not present on microarray. Nodes with a single border represent individual components (at gene or protein level). Nodes with a double border represent families of related components and/or complexes containing multiple individual components. Double bordered nodes may have a mixture of different colors due to different microarray expression levels for each of their individual components. Colors represent microarray determined RNA expression levels.

The top molecular and cellular functions, as determined by IPA (Ingenuity Pathway Analysis), enriched by these differentially expressed genes in either the WT or KO groups were virtually the same (Table 1). The biological functions enriched in the top IPA interaction network for the WT group (Figure 11A) are: Cell Cycle, Cellular Assembly and Organization, DNA Replication, Recombination, and Repair, which are the same as in the top network for the KO group (Figure 11B). Among the top 25 regulated genes in the WT group, 14 of them were found in the KO group (Table 2).

**Table 1:** WT and KO groups share the same top molecular and cellular functions (determined from IPA) of gene expression changes in the lung, induced by naphthalene at day 7

Molecular and Cellular Functions	WT			KO		
	*p*-value	# Molecules	Rank	*p*-value	# Molecules	Rank
Cell Cycle	1.08E-23-4.26E-03	96	1	2.73E-25-2.47E-03	99	1
Cellular Assembly and Organization	1.08E-23-4.26E-03	59	2	2.73E-25-2.47E-03	83	2
DNA Replication, Recombination, and Repair	1.08E-23-4.26E-03	52	3	2.73E-25-2.03E-03	73	3
Cellular Movement	1.11E-10-1.31E-03	26	4	2.06E-12-2.27E-03	94	4
Cellular Function and Maintenance	3.41E-07-4.26E-03	29	5	7.85E-09-2.27E-03	90	6
Cell Death and Survival	8.15E-07-3.96E-03	119	6	2.22E-10-1.26E-03	125	5

**Table 2:** Similarity of top 25 genes regulated by naphthalene treatment at day 7 between WT and KO groups

WT				KO			
Gene Symbol	Entrez ID	Fold	FDR	Gene Symbol	Entrez ID	Fold	FDR
Clca1	23844	27.219	0.00010	Clca1	23844	45.604	0.00003
Agr2	23795	7.942	0.00015	Chil4	104183	29.363	0.01534
Fgg	99571	7.192	0.00007	Agr2	23795	8.171	0.00012
Muc5ac	17833	5.142	0.00583	Cd177	68891	7.281	0.00003
Gp2	67133	4.911	0.00019	Retnla	57262	5.324	0.00011
Retnla	57262	4.788	0.00016	Igkv5–43	381783	−5.238	0.00643
Tff2	21785	4.784	0.00323	Muc5ac	17833	5.049	0.00701
Reg3g	19695	4.016	0.00911	Ghrl	58991	4.906	0.00001
Ighv1–34	628614	−3.987	0.01838	Fgg	99571	4.545	0.00033
Cd177	68891	3.897	0.00036	Gp2	67133	4.362	0.00027
Slc26a4	23985	3.812	0.00255	Igkv6–14	667881	−4.055	0.04842
Cdk1	12534	3.643	0.00010	Igkv9–124	243431	−3.883	0.00417
Muc5b	74180	3.562	0.00280	Fmo3	14262	−3.879	0.00047
Mfsd2a	76574	3.491	0.00013	Slc26a4	23985	3.824	0.00273
Sptssb	66183	3.426	0.00022	Fkbp5	14229	3.793	0.00018
Fcgbp	215384	3.360	0.04863	Fcgbp	215384	3.787	0.03450
Saa3	20210	3.320	0.02606	Cdk1	12534	3.781	0.00007
Ghrl	58991	3.265	0.00007	Sptssb	66183	3.630	0.00014
Ccna1	12427	3.192	0.00006	Pbk	52033	3.567	0.00025
Ect2	13605	3.140	0.00015	9530077C05Rik	68283	3.491	0.00006
Pbk	52033	3.138	0.00054	Zbtb16	235320	3.446	0.00673
Top2a	21973	3.102	0.00016	Fabp1	14080	−3.367	0.00070
Oit1	18300	3.043	0.00527	Mfsd2a	76574	3.344	0.00013
Prc1	233406	3.035	0.00055	Pla2g4c	232889	3.318	0.02976
BC048546	232400	2.972	0.04711	Ccna2	12428	3.309	0.00029

Underline indicates common genes for WT and KO. Italic indicates decreased expression in treated vs. untreated mice

**Figure 12:**
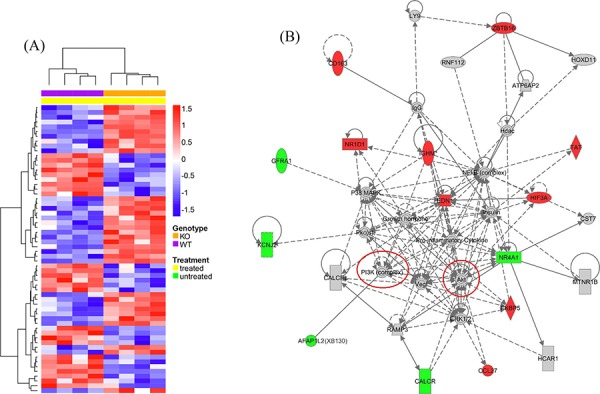
Microarray determined gene expression differences between KO and WT mice 7 days after naphthalene treatment **A.** Hierarchical clustering heatmap shows relative expression differences for each of 60 genes with significant expression differences between KO and WT mice 7 days after naphthalene treatment. See details in Figure 10 legends. **B.** The PI3K/Akt signal pathway is related to genes differentially expressed between KO and WT mice in the lung 7 days after naphthalene treatment, as shown by the top IPA generated interaction network. The biological functions enriched by the molecules in this network are Tissue Morphology, Cell Mediated Immune Response and Cellular Development. Red: up-regulation (KO relative to WT). Green: down-regulation. Red circles: PI3K/Akt pathway components are highlighted. See Figure 11 legend for other details.

We also directly compared the gene expression between KO and WT naphthalene treated animals: 60 genes were found to have significantly different expression levels (Figure 12A). The biological functions enriched by the top IPA generated interaction network (Figure 12B) are Tissue Morphology, Cell Mediated Immune Response and Cellular Development. A group of genes involved in inflammatory and immune responses are up-regulated in KO mice in comparison to those in WT mice, including CD163 (a member of the scavenger receptor cysteine-rich superfamily, expressed in monocytes and macrophages), EDN1 (endothelin 1, a potent vasoconstrictor), FKBP5 (FK506 binding protein 5, which plays a role in immunoregulation), and HIF3A (hypoxia inducible factor 3, alpha subunit). Moreover, this network contains many interactions involving members of several intracellular signal transduction pathways, especially the PI3K/Akt pathway (Figure 12, highlighted by red circles), consistent with our observation of XB130 phosphorylation of members of this pathway (Figure 9).

To find pathways activated by naphthalene treatment that differ significantly in their activity between KO and WT mice, we performed a canonical pathway activation z-score comparison in IPA of naphthalene treated vs. untreated mice in KO compared to WT groups. The canonical pathway that had the largest difference in predicted activation between KO and WT was Cyclins and Cell Cycle Regulation. Although this pathway was predicted to be activated in both groups, it was predicted to have higher activity in WT compared to KO mice.

Expression of multiple cyclins and CDK1 (cyclin-dependent kinase 1) were significantly upregulated in both groups. However, CDKN1A (cyclin-dependent kinase inhibitor 1A (p21, Cip1) and WEE1 (WEE1 G2 checkpoint kinase) were only significantly increased in the KO group (Table 3). IPA's Molecule Activity Predictor suggests that upregulation of p21 and WEE1 can inhibit cell cycle progression at G1, S and G2 phases (Figure E2 in the online data supplement), which may help to explain why XB130 deficiency delays cell proliferation and repair.

**Table 3:** Naphthalene-induced gene expression changes in the pathway of Cyclins and Cell Cycle Regulation are similar between the KO and WT groups, except for CDKN1A and WEE1, which are significantly upregulated only in the KO group

Gene symbol	Gene name	KO		WT	
		Fold change	FDR	Fold change	FDR
CCNA1	cyclin A1	2.310	2.93E-03	3.168	8.72E-04
CCNA2	cyclin A2	3.057	3.69E-03	2.404	1.08E-02
CCNB1	cyclin B1	2.494	2.49E-02	2.068	4.87E-03
CCNB2	cyclin B2	2.510	4.45E-03	2.272	7.31E-03
CCNE2	cyclinE2	1.808	5.67E-03	1.647	1.14E-02
CDK1	cyclin-dependent kinase1	3.419	1.41E-03	3.409	1.64E-03
CDKN1A	cyclin-dependent kinaseinhibitor 1A (p21, Cip1)	1.524	4.35E-02	1.294	2.08E-01
WEE1	WEE1 G2 checkpoint kinase	1.604	7.75E-03	1.195	2.63E-01

## DISCUSSION

Proliferation of BASCs and epithelial cells after injury is crucial for bronchial epithelial repair and regeneration. In this study, we showed that the presence of XB130 in bronchial Club cells promotes proliferation of BASCs and Club cells in the small airways during the repair process after naphthalene challenge. These functions are likely mediated through the PI3K/Akt pathway. Moreover, microarray data suggest that XB130 deficiency may cause activation of p21 and WEE1 related inhibition of cell cycle progression and cell proliferation.

At the early injury phase, cell death score and TUNEL assay did not show significant differences between WT and *XB130* KO mice. Naphthalene induces severe death of Club cells [3–5]. The presence of XB130 in Club cells may have limited protective effects on cell survival. It will be interesting to see under other pathological conditions, such as sepsis, ventilator-induced lung injury, cigarette smoking, air pollution, or allergic responses, whether XB130 would have protective effects on cell survival.

On the other hand, at days 7 and 14, which is the repair phase, H&E staining showed that recovery of injured bronchial epithelium was delayed in *XB130* KO mice. Moreover, in KO mice, the total cell numbers at the BADJs, the number of Club cells, and the ratio of CCSP+ to β-tubulin IV+ cells were also significantly lower. XB130 was mainly found in the CCSP+ cells, not in the ciliated cells. Therefore, XB130 deficiency mainly affected proliferation of Club cells.

Migration of surviving cells after injury is the initial response to cover the wound in airway epithelial repair [1]. Park *et al*. studied the behavior of ciliated cells after naphthalene treatment, and showed that epithelial integrity was initially maintained by extension and migration of squamous cells in a process that proceeded cell proliferation [25]. Kida *et al*. indicated that cell migration was involved in the airway epithelial repair process [23]. XB130 was involved in cell migration through Rac- and cytoskeletal-dependent mechanisms [14]. In the present study, however, we did not see significant differences in terms of cell spreading and migration. IF staining showed that XB130 was not stained in ciliated cells in WT mice. Therefore, the absence of XB130 may not affect the spreading and migration of ciliated cells. We have recently studied the role of XB130 in large airway epithelial repair and regeneration with an isogenic murine tracheal transplant model, and we found that the major function of XB130 in trachea is to influence the differentiation of the airway epithelium [20]. It is possible that the same protein may play different roles in different cell types based on its expression levels and the differentiation status of the cells.

Variant Club cells are progenitor cells located at the BADJs that proliferate and differentiate into regular Club cells after airway epithelial injury [6]. Regenerating Club cells may give rise to cell types resembling those seen in normal bronchioles [31]. At day 5, in WT mice, the number of BASCs was significantly higher than at days 0 and14 and in the control group. In *XB130* KO mice, even though the number of BASCs was also increased at days 5 and 7, there were no significant differences among time points. Furthermore, at day 5, the number of proliferative (Ki-67+) epithelial cells was significantly lower in KO mice. Thus, the slowdown of the airway epithelial repair and regeneration in XB130 may be due to less proliferation of BASCs at BADJs.

In the present study, there was much less staining of p-p85α, p-Akt and p-GSK-3β in airway epithelial cells in *XB130* KO mice at day 7. The PI3K/Akt pathway can further regulate β-catenin in airway epithelial repair after naphthalene treatment [30]. Akt can inhibit GSK-3β, therefore preventing the degradation of β-catenin. Akt can also phosphorylate β-catenin directly on Ser552 for its translocation to the nucleus [32]. Therefore, reduced phosphorylation of PI3K/Akt/GSK-3β in Club cells may affect cell proliferation and airway repair and regeneration. Our microarray/bioinformatics study further supports the involvement of the PI3K/Akt pathway in XB130 related cellular responses (Figure 12).

The microarray data also suggested more inflammatory activities in the KO mice. The naphthalene-induced expression of CDKN1A (p21, Cip1) and WEE1 was only up-regulated in the KO mice, which may inhibit cell proliferation and cell cycle progression (Figure E2), and subsequently delay repair and regeneration of airway epithelial cells. Because whole lung RNA was used for microarray analysis, we cannot specify which cell type(s) are responsible for the major observed changes in gene expression. However, this analysis helps to determine the overall changes of gene expression in the lung. The changes that we highlighted are consistent with our other studies.

In conclusion, our results indicate that XB130 is involved in BASC and Club cell proliferation. We speculate that this function is mainly mediated through the phosphorylation of PI3K/Akt/GSK-3β and related signaling events. Clinically relevant models related to small airway diseases should be used to further explore the role of XB130 in airway epithelial repair, regeneration and remodeling. Moreover, the role of XB130 in airway repair should be addressed directly in human lung diseases.

## MATERIALS AND METHODS

More detailed materials and methods are presented as online Supplementary Materials.

### Animal care

The study protocol was approved by Animal Use and Care Committee of the University Health Network. All animals received humane care in compliance with the Guide for the Care and Use of Experimental Animals formulated by the Canadian Council on Animal Care.

*XB130* KO mice were generated in collaboration with Dr. Tak W. Mak (University of Toronto), and backcrossed more than 10 generations on a C57BL/6 genetic background. *XB130* KO mice showed a normal life span, and did not have obvious phenotypes in a series of physiological tests compared to their wild-type (WT) littermates [20].

### Naphthalene treatment

Female *XB130* KO mice and their female WT littermates (8 - 12 weeks of age) were either treated with naphthalene (Sigma-Aldrich, St. Louis, MO) dissolved in corn oil (Sigma-Aldrich) at a dose of 200 mg/kg body weight intraperitoneally, or were untreated. Mice were sacrificed at days 0, 2, 5, 7 and 14 after treatment. Corn oil alone (0.25 ml/kg) was administered to a control group, where mice were sacrificed at day 2 after corn oil treatment [33]. For each time point, 4 - 6 mice were used per group. Lung tissues were collected for subsequent studies.

### Histological study

The left lung tissue sections were stained with hematoxylin and eosin (H&E). Cell death was determined semi-quantitatively from 10 randomly chosen terminal bronchi (x400) [33]. Epithelial damage was expressed as a ratio between denuded and total surface within the BADJ (200 μm) [6]. ImageJ (1.46r) (NIH, Bethesda, MD) was used for data analyses.

### Immunohisotochemistory (IHC) and immunofluorescence (IF)

The primary antibodies used are shown in Table E1 (in the online data supplement). IHC was performed using a Vectastain ABC kit (Vector Laboratories, Burlington, ON). All fluorescent staining was performed with appropriate secondary antibodies from Invitrogen and mounted with Prolong Gold Antifade Mountant with DAPI^®^ (Invitrogen, Burlington, ON). Positive cells were counted from at least 5 BADJs (x400).

### TUNEL assay

Terminal transferase dUTP nick end labelling (TUNEL) was performed with *In Situ* Cell Death Detection Kit, TMR red (Roche, Penzberg, Germany). We randomly chose at least 10 fields (x400) per slide to quantify TUNEL+ epithelial cells attached on basement membrane, expressed as percentage of total DAPI+ cells [34].

### Quantitative RT-PCR

Total RNA was extracted from the right lung. Quantitative RT-PCR was performed [17]. PCR primers are shown in Table E2 (in the online data supplement).

### Microarray

Lung tissue RNA from day 7 after naphthalene treatment or from untreated WT and KO mice (*n* = 4/group) were used with Mouse Gene ST 2.0 chips (Affymetrix, Santa Clara, CA) and microarray data were analyzed as described [35]. See details in the online supplement.

### Statistical analysis

All values are expressed as mean ± standard deviation (SD). Statistical analyses were performed by using Student's *t*-test and analysis of variance (ANOVA) with GraphPad Prism 5.0 (GraphPad, La Jolla, CA). *p* < 0.05 was considered to be statistically significant.

## SUPPLEMENTARY DATA FIGURES AND TABLES


