# Supplementary material for: XB130 promotes bronchioalveolar stem cell and Club cell proliferation in airway epithelial repair and regeneration
Source: Oncotarget. 2015 Sep 3;6(31):30803–17. doi: 10.18632/oncotarget.5062 (PMC4741569; doi:10.18632/oncotarget.5062)
Supplement: Supplementary file 1 [file oncotarget-06-30803-s001.pdf]

SUPPLEMENTARY DATA

Animal care

The study protocol was approved by the Animal Use and Care Committee of the University Health Network. All animals were housed in a pathogen-free facility, with a 12 h light-dark cycle. They were provided free access to water and food, and received humane care in compliance with the Guide for the Care and Use of Experimental Animals formulated by the Canadian Council on Animal Care (Ottawa, ON, Canada).

XB130 KO mice were generated in collaboration with Dr. Tak W. Mak (University of Toronto), and backcrossed more than 10 generations on a C57BL/6 genetic background. XB130 KO mice showed a normal life span and did not have obvious phenotypes in a series of physiological testing compared to wild-type (WT) littermates [1].

Naphthalene treatment

Female XB130 KO mice and their female WT littermates (8–12 weeks of age) were used. Naphthalene (Sigma-Aldrich, St. Louis, MO) was dissolved in corn oil (Sigma-Aldrich) and administrated at a dose of 200 mg/kg body weight. Naphthalene or corn oil alone (0.25 ml/kg body weight) was administered as a single intraperitoneal injection [2]. Mice were sacrificed at days 0, 2, 5, 7 and 14 after naphthalene treatment. In a control group, mice were sacrificed at day 2 after treatment with corn oil alone. At each time point, 4 - 6 mice were used per group. Lung tissues were collected for subsequent studies.

Histological study

After sacrifice, the lungs were harvested, and the left lung was fixed with 10% buffered formalin for histological studies, and the remaining lung tissue was snap-frozen and stored at -80°C for RNA extraction. The lung tissues were embedded in paraffin and cut at 5 μm thickness, and the sections were stained with hematoxylin and eosin (H&E). Images were captured using Olympus BX51-FL (Olympus Co, Ltd). We randomly chose 10 terminal bronchi (imaged at x400) from each lung to determine the extent of cell death and epithelial damage. Cell death was estimated semi-quantitatively by using the criteria described by Oliver *et al* [2]. Briefly, 0: no death; 1: mild (occasional detection); 2: moderate (more frequent); 3: severe (very frequent). Epithelial damage was expressed as a ratio between nude surface and total surface [3] within BADJ (200 μm) as defined [4] by measuring the airway lumen with ImageJ (1.46r) (NIH, Bethesda, MD).

Immunohisotochemistory (IHC) and immunofluorescence (IF)

After deparaffinization, sections were incubated in 10 mM citric buffer (pH 6) and heated for 20 minutes and cooled down to room temperature for 20 minutes, and then incubated with 3% H_2O_2 for 30 minutes to block endogenous peroxidase activity for IHC. After blocking, sections were incubated with primary antibodies. The primary antibodies used are listed in Table E1. Then, sections were incubated with appropriate secondary antibodies. IHC was performed using a Vectastain ABC kit (Vector Laboratories, Burlington, ON) with 3-3-diaminobenzidine as chromogen, and sections were counterstained with hematoxylin, and images were captured via Olympus BX51-FL. For IF, the secondary antibodies used were: donkey anti-goat Alexa Fluor® 488, donkey anti-rabbit Alexa Fluor® 555 and goat anti-mouse Alexa Fluor® 555 (1:200, Invitrogen, Burlington, ON), and sections were mounted with Prolong Gold Antifade Mountant with DAPI® (Invitrogen). The slides were examined with an Axiovert 200M microscope (Zeiss, Oberkochen, Germany) and Olympus BX-51, and images were captured via CoolSnap HQ camera (Roper, Ottobrunn, Germany) and QImaging colour camera (Olympus Co, Ltd). We randomly chose at least 5 BADJs (imaged at x400) and counted immune-positive cells.

TUNEL assay

Cell Death was assessed by *in* situ terminal transferase dUTP nick end labeling (TUNEL) with *In Situ* Cell Death Detection Kit, TMR red (Roche, Penzberg, Germany). We randomly chose at least 10 fields (x400) per slide and quantified TUNEL-positive epithelial cells attached on basement membrane using ImageJ (1.46r). The percentage of labeled cells was expressed by TUNEL-positive cells/DAPI-positive cells x 100% [5].

Quantitative RT-PCR

Total RNA was extracted using RNeasy kit (Qiagen, Duesseldorf, Germany). cDNA was synthesized from total RNA using MuLV reverse Transcriptase (Invitrogen). Quantitative RT-PCR was performed using SYBR Green I master PCR kit on Light Cycler480 (Roche Diagnostics). Each assay included a standard curve of six serial dilutions and no-template negative control. All assays were performed in triplicate. The primers used are shown in Table E2. The relative expression level of each target gene was calculated after normalization with GAPDH.

Microarray and data analysis

Total RNA was extracted using RNeasy kit (Qiagen, Duesseldorf, Germany) from total lung tissue at day 7 after naphthalene treatment or from untreated controls of WT and KO mice ($n = 4$ per group). Mouse Gene ST 2.0 chips from Affymetrix (Santa Clara, CA) were used. Hybridization and scanning was performed by The Centre for Applied Genomics, The Hospital for Sick Children, Toronto, Canada.

Differential gene expression analysis. Raw data from Affymetrix CEL files were pre-processed using RMA in R [6]. To reduce ambiguities from multiple probesets per gene, we utilized the version 19 custom CDF annotation package published by the Molecular and Behavioral Neuroscience Institute Microarray Lab at the University of Michigan [7]. This produced a unique expression level per gene after RMA pre-processing. Statistically significant differential expression was determined using the limma package [8]. The limma model included 4 contrasts to test pairwise differences between KO vs. WT or naphthalene treated vs. untreated mice. *P* values for each contrast were adjusted for multiple comparison utilizing the Benjamini-Hochberg false discovery rate correction (FDR). Genes were considered to be significantly differentially expressed if both $|\text{fold change}| \geq 1.5$x and FDR ≤ 0.05.

IPA pathway analysis. Affymetrix CEL files were imported into Partek Genomics Suite 6.6 software (Partek Inc., St. Louis, MO) and processed using RMA at the probeset level. Probeset differential expression was determined using Partek's Analysis of Variance (ANOVA) function. Multiple test correction and selection of significant features were performed as described above. The ANOVA results were imported into QIAGEN's Ingenuity® Pathway Analysis (IPA®, QIAGEN Redwood City, http://www.qiagen.com/ingenuity), for pathway, network and functional analyses.

Statistical analysis

All values are expressed as mean ± standard deviation (SD). Statistical analyses were performed by using Student's *t*-test and analysis of variance (ANOVA) with GraphPad Prism 5.0 (GraphPad, La Jolla, CA). $P < 0.05$ was considered to be statistically significant.

REFERENCES

1. Zhao J, Wang Y, Wakeham A, Hao Z, Toba H, Bai X, Keshavjee S, Mak TW, Liu M. XB130 deficiency affects tracheal epithelial differentiation during airway repair. PLoS One. 2014; 9:e108952.
2. Oliver JR, Kushwah R, Wu J, Cutz E, Yeger H, Waddell TK, Hu J. Gender differences in pulmonary regenerative response to naphthalene-induced bronchiolar epithelial cell injury. Cell Prolif. 2009; 42:672–687.
3. Tiozzo C, De Langhe S, Yu M, Londhe VA, Carraro G, Li M, Li C, Xing Y, Anderson S, Borok Z, Bellusci S, Minoo P. Deletion of Pten expands lung epithelial progenitor pools and confers resistance to airway injury. Am J Respir Crit Care Med. 2009; 180:701–712.
4. Giangreco A, Reynolds SD, Stripp BR. Terminal bronchioles harbor a unique airway stem cell population that localizes to the bronchoalveolar duct junction. Am J Pathol. 2002; 161:173–182.
5. Linnoila RI, Jensen-Taubman S, Kazanjian A, Grimes HL. Loss of GFI1 impairs pulmonary neuroendorine cell proliferation, but the neuroendocrine phenotype has limited impact on post-naphthalene airway repair. Lab Invest. 2007; 87:336–344.
6. R Core Team. R: A language and environment for statistical computing. R Foundation for Statistical Computing. 2015; http://www.R-project.org.
7. Dai M, Wang P, Boyd AD, Kostov G, Athey B, Jones EG, Bunney WE, Myers RM, Speed TP, Akil H, Watson SJ, Meng F. Evolving gene/transcript definitions significantly alter the interpretation of GeneChip data. Nucleic Acids Res. 2005; 33:e175.
8. Ritchie ME, Phipson B, Wu D, Hu Y, Law CW, Shi W, Smyth GK. limma powers differential expression analyses for RNA-sequencing and microarray studies. Nucleic Acids Res. 2015; 43:e47.

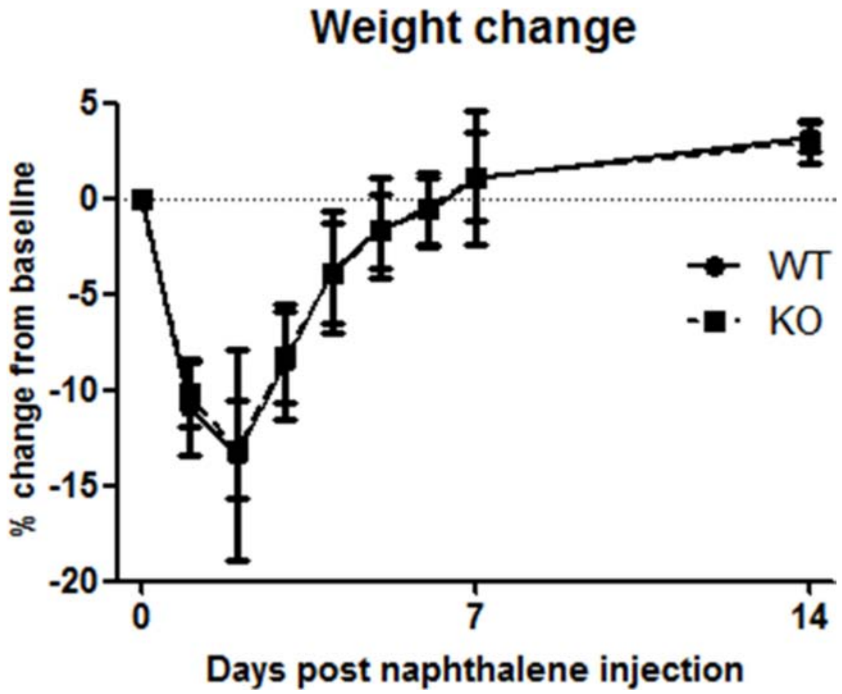

Supplementary Figure S1: The absence of XB130 did not affect the naphthalene-induced body weight loss and recovery. WT = Wild type, KO = XB130 knock out.

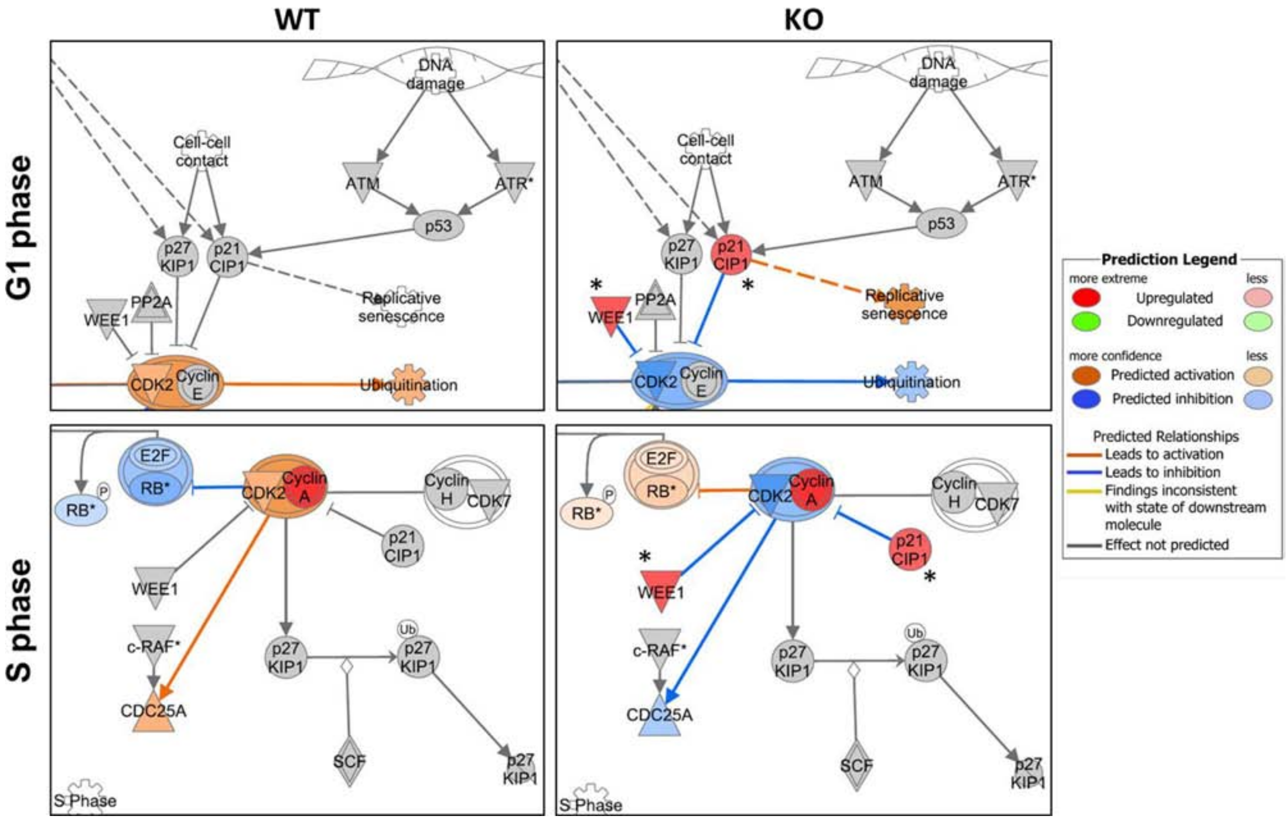

Supplementary Figure S2: The pathway of Cyclins and Cell Cycle Regulation is differentially regulated by naphthalene treatment between the WT and KO mice. Molecule Activity Predictor (MAP) analysis was performed in IPA to predict the upstream and/or downstream effects of cyclins and related genes. Up-regulation of CDKN1A (p21 CIP1) and WEE1 in the KO mice only (asterisks) may inhibit cyclin related cell cycle progression.

Supplementary Table S1: Primary antibodies used for immunohistochemistry and immunofluorescent staining

Antigen	Host	Source	Dilution
XB130	rabbit	Abgent	1:1000
Ki-67	rabbit	Lab Vison	1:100
CCSP	rabbit	Upstate	1:10000
	goat	Santa Cruz Biotechnology	1:500
β-tubulin IV	mouse	Sigma-Aldrich	1:100
Sftpc	rabbit	Seven Hills Bioreagents	1:1000
phospho-P85α-Tyr508	rabbit	Santa Cruz Biotechnology	1:250
phospho-AKT-Ser473	rabbit	Cell Signaling	1:50
phospho-GSK-3β-Ser9	rabbit	Cell Signaling	1:500

Supplementary Table S2: PCR Primers used in the present study

Gene	Direction	Sequence (5′ - 3′)
XB130	Forward	TCAGCATCTCCAGAC
	Reverse	GGCTGTTTCCTCTCT
CCSP	Forward	ATGAAGATCGCCATCACAATCAC
	Reverse	GGATGCCACATAACCAGACTCT
β-tubulin IV	Forward	AACCCGGCACCATGGACTCTGT
	Reverse	TGCCTGCTCCGGATTGACCAAATA
Foxj1	Forward	CCCTGACGACGTGGACTATG
	Reverse	GCCGACAGAGTGATCTTGGT
Wnt7b	Forward	CTTCACCTATGCCATCACGG
	Reverse	TGGTTGTAGTAGCCTTGCTTCT
Fgf10	Forward	TTTGGTGTCTTCGTTCCCTGT
	Reverse	TAGCTCCGCACATGCCTTC
β-catenin	Forward	CCCAGTCCTTCACGCAAGAG
	Reverse	CATCTAGCGTCTCAGGGAACA
Snail1	Forward	CACACGCTGCCTTGTGTCT
	Reverse	GGTCAGCAAAAGCACGGTT
